# The CMV-Specific CD8^+^ T Cell Response Is Dominated by Supra-Public Clonotypes with High Generation Probabilities

**DOI:** 10.3390/pathogens9080650

**Published:** 2020-08-13

**Authors:** Kilian Schober, Pim Fuchs, Jonas Mir, Monika Hammel, Lorenzo Fanchi, Michael Flossdorf, Dirk H. Busch

**Affiliations:** 1 Institute for Medical Microbiology, Immunology and Hygiene, Technische Universität München (TUM), 81675 Munich, Germany; jonas.mir@tum.de (J.M.); monika.hammel@tum.de (M.H.); michael.flossdorf@tum.de (M.F.); 2 German Center for Infection Research (DZIF), partner site Munich, Munich, Germany; 3 ENPICOM B.V., 5211 AX ‘s-Hertogenbosch, The Netherlands; p.fuchs@enpicom.com (P.F.); l.fanchi@enpicom.com (L.F.)

**Keywords:** memory inflation, public clonotypes, generation probability, T cell response, TCR, convergent recombination, TCR repertoire

## Abstract

Evolutionary processes govern the selection of T cell clonotypes that are optimally suited to mediate efficient antigen-specific immune responses against pathogens and tumors. While the theoretical diversity of T cell receptor (TCR) sequences is vast, the antigen-specific TCR repertoire is restricted by its peptide epitope and the presenting major histocompatibility complex (pMHC). It remains unclear how many TCR sequences are recruited into an antigen-specific T cell response, both within and across different organisms, and which factors shape both of these distributions. Infection of mice with ovalbumin-expressing cytomegalovirus (IE2-OVA-mCMV) represents a well-studied model system to investigate T cell responses given their size and longevity. Here we investigated > 180,000 H2k^b^/SIINFEKL-recognizing TCR CDR3α or CDR3β sequences from 25 individual mice spanning seven different time points during acute infection and memory inflation. In-depth repertoire analysis revealed that from a pool of highly diverse, but overall limited sequences, T cell responses were dominated by public clonotypes, partly with unexpectedly extreme degrees of sharedness between individual mice (“supra-public clonotypes”). Public clonotypes were found exclusively in a fraction of TCRs with a high generation probability. Generation probability and degree of sharedness select for highly functional TCRs, possibly mediated through elevating intraindividual precursor frequencies of clonotypes.

## 1. Introduction

T cells play a crucial role in the control of chronic and latent infections. Latent infection with CMV affects 60–100% of the general population [1]—largely depending on the socioeconomic status—but does not cause any symptoms unless the immune system is compromised. In immunosuppressed patients, for example hematopoietic stem cell transplantation recipients, CMV is indeed a major cause of morbidity and mortality [2]. The significance of T cell mediated protection from overt CMV infection is further strongly supported by adoptive transfer experiments in mice [3,4] and through the success of adoptive T cell therapy in human patients [5,6].

In immunocompetent individuals, viral latency and immune control seem to be in equilibrium. On the T cell side, this equilibrium is maintained by antigen-specific populations that can become extremely large (“inflationary T cells”) [7,8]. Inflationary T cells often harbor a senescent phenotype [9] and an overall skewed CD4/CD8 ratio. Such a CMV-induced so-called immune risk phenotype [10] has been associated with increased mortality in the elderly [11,12,13]. Overall, the protective importance, size and longevity of CMV-specific T cells raise an intriguing question—at what cost does the immune system dedicate so much of its capacity to target individual CMV antigens [14]? Furthermore, however, these aspects have also made CMV T cell immunity an attractive model system to investigate acute and chronic T cell responses in general.

Since CMV-specific T cell responses have been studied so deeply, a surprising finding became apparent early on: CMV-specific T cell populations often contain public clonotypes, i.e., T cell clones that occur in more than a single individual [15,16,17]. This is astonishing as it is a canonical feature of the adaptive immune system to be programmed by non-self antigens only and the TCR recombination process has been long believed to be random. In order to mediate robust protection against a plethora of different and unknown foreign antigens, a highly diverse TCR repertoire is needed [18]. V(D)J recombination, deletions and additions of individual nucleotides in the CDR3 region of the TCR variable chains, as well as differential pairing of α and β chains, yield a theoretical diversity of up to 10^20^ or in some estimates even 10^61^ different solutions for TCRs [19,20]. The actual diversity in a single mouse [19] or human being [21] is considerably lower, but still believed to be in the range of about 10^7^–10^8^ unique TCRs. It remains largely unanswered how many TCRs fit to a given antigen in principle (across different individuals) and how many TCRs contribute to an antigen-specific T cell response in a single individual.

If the number of structural solutions of a TCR to a given epitope is finite, it is intuitive that clonotypes would re-occur, rendering them public. Even more importantly, the theoretical TCR repertoire is in reality highly skewed, since generation probabilities for individual TCRs are highly heterogenous (“TCR generation bias”) [20,22]. Indeed, it has been experimentally shown that the degree of sharedness of public clonotypes is associated with its generation probability. This phenomenon of high generation probabilities leading to public clonotypes has been termed “convergent recombination” [17,22,23,24].

While there are indications that public clonotypes are found disproportionately often in CMV antigen-specific T cell responses, few studies have investigated the degree of “degree of sharedness”, i.e., in how many different individuals do public clonotypes re-occur [23]. Further, it is unclear how dominant such public clonotypes are within CMV-specific immune responses. A recent study showed that public clonotypes are dominant in inflationary CMV-specific T cell populations, but this work only investigated very late time points of infection, did not take into account different degrees of sharedness and calculated generation probability merely by distance from germline [25]. In recent years, computation of generation probabilities became significantly more refined and can now also take into account biochemical features of CDR3 sequences, such as hydrophobicity of amino acids at certain positions or common motifs [26,27,28].

We have previously performed in-depth repertoire sequencing of CMV antigen-specific T cell populations from 25 mice spanning seven different time points during acute infection with murine CMV and memory inflation [29]. We now further investigated the obtained 42,154 CDR3α and 139,374 CDR3β sequences with respect to their public occurrence (including quantitative analysis of the degree of sharedness between individual mice) and their generation probability. This analysis revealed that while there is an astonishingly high overall number of unique CDR3 sequences within the antigen-specific pool, dominant clonotypes are highly enriched for public clones, most notably clones with very high degrees of sharedness (“supra-public clonotypes”) and high generation probabilities. While it remains an intriguing speculation that the TCR recombination machinery may “foresee” which specificities would be highly useful in the fight against pathogens [27], we hypothesize that robust recruitment may simply be a result of higher precursor numbers in the antigen-unexperienced repertoire.

## 2. Results

**Figure 1:**
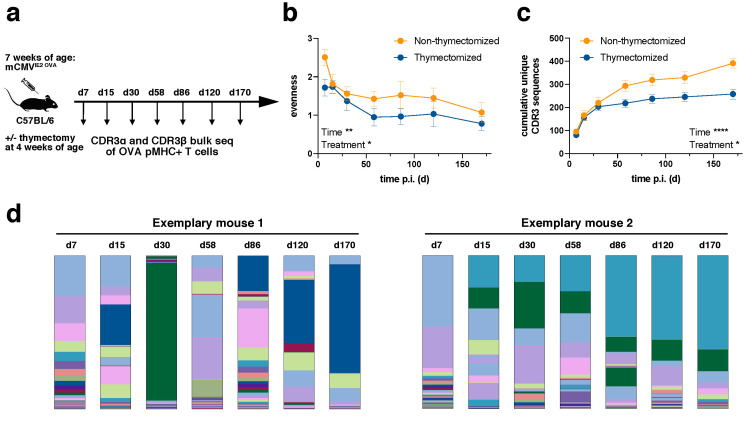
TCR repertoire evolution of H2k^b^/SIINFEKL-specific T cells during CMV infection. (**a**) Experimental setup; (**b**) evenness of TCR repertoires based on CDR3β sequences (analogous results were obtained for CDR3α). Higher values indicate homogenously distributed repertoires whereas lower values indicate skewed repertoires. Shown is the mean +/− SEM for *n* = 5–7 mice per treatment (thymectomy vs. no thymectomy). Representative of two independent experiments. Statistical testing by mixed-effects model (REML) analysis for fixed effects (type Ⅲ), * *p* value < 0.05, ** *p* value < 0.01; (**c**) cumulative unique CDR3β sequences of TCR repertoires per mouse (analogous results were obtained for CDR3α). Shown is the mean +/− SEM for *n* = 5–7 mice per treatment. Representative of two independent experiments. Statistical testing by two-way repeated measures (RM) ANOVA analysis. * *p* value < 0.05, **** *p* value < 0.0001; (**d**) TCR repertoire evolution in two representative mice (both representative mice are from the thymectomized groups, but show overall repertoire evolution patterns that are representative of thymectomized as well as non-thymectomized mice). Different colors denote unique CDR3β sequences (analogous results were obtained for CDR3α).

We infected C57BL/6 mice intraperitoneally (i.p.) with a tissue culture-derived Smith mCMV strain expressing OVA under the IE2 promotor (IE2-OVA-mCMV, Figure 1a), which led to inflationary T cell responses against OVA [30]. All mice were infected at the age of seven weeks. Half of the mice had been previously thymectomized at the age of four weeks in order to investigate how the TCR repertoire evolved from a narrower precursor repertoire and deduct the influence of recent thymic emigrants on the inflationary T cell population. The success of thymectomy was controlled by qPCR for TCR excision circles [29]. All mice were distributed across two independent experimental cohorts (two non-thymectomized cohorts, two thymectomized cohorts). We sorted the H2k^b^/SIINFEKL-specific CD8+ T cell population from peripheral blood by flow cytometry at day 7, 15, 30, 58, 86, 120 and 170 post infection with sort purities of at least 99.5% [29]. Following RNA extraction and rapid amplification of cDNA ends (RACE) PCR, CDR3α and CDR3β bulk sequencing was performed. Sequences were considered only if an intact unique molecular identifier (UMI) was present and found more than once in a given sample. Clonotypes were defined as chains with identical CDR3 amino acid sequences. For further details see Materials and Methods and references [29].

### 2.1. TCR Repertoire Evolution Stabilizes during Memory Inflation

Diversity can be assessed with a focus on the evenness of the distribution or the richness of unique samples. In relation to TCR repertoires, a repertoire can be diverse when TCR clones are evenly distributed despite a low number of unique samples or it can be diverse in terms of richness with a high number of unique samples even though samples are distributed in a very skewed manner. We first evaluated the evenness of TCR repertoires (Figure 1b). The evenness in both non-thymectomized and thymectomized animals decreased over time. The greatest loss in diversity was observed during the first 60 days, whereas thereafter the evenness stayed fairly stable. This is consistent with the outgrowth of stable dominating clonotypes during memory inflation [29]. The evenness in thymectomized animals was generally lower than in non-thymectomized animals. This difference was already apparent at day 7 post infection. Since thymectomy took place three weeks before infection, this could indicate that a narrower precursor repertoire in thymectomized animals is driven into more accentuated clonal selection early on. To investigate this more closely, we analyzed the cumulative number of unique CDR3β sequences over time for each mouse (Figure 1c). The number of unique clones was only slightly elevated in non-thymectomized compared to thymectomized mice in the early phase of the immune response, but a richer repertoire was maintained throughout the chronic phase of infection. The decreased evenness in thymectomized animals can therefore not be simply explained by a narrower precursor repertoire in terms of unique clones. The decreased richness in thymectomized animals over time speaks for less efficient recruitment of new antigen-specific T cells. This could argue for recruitment of naïve T cells into the inflationary T cell response in non-thymectomized animals, but would also be in line with a defect of nourishment from a pool of memory T cells only becoming apparent at later time points of the immune response [31]. The repertoire evolution with regards to evenness, richness and qualitative clonal succession [29] can be seen in two representative mice (Figure 1d).

The plateau of unique CDR3 sequences at late time points indicates a limited absolute number of antigen-specific unique clonotypes within a given mouse (Figure 1c). In an individual non-thymectomized mouse, we found around 200 unique CDR3α (data not shown) and around 400 CDR3β (Figure 1c) sequences on average. These numbers were reduced in thymectomized mice. It is likely that these numbers represent an underestimate of the absolute number of antigen-specific clonotypes within a mouse. Two important reasons for this are the fact that a given CDR3β sequence can differentially pair with CDR3α sequences and that repertoire analysis was conducted from a small fraction (around 100 µl) of peripheral blood. The confounding effect of the sampling bias should be, however, alleviated through repetitive sampling over time (Figure 1c). Importantly, the plateau of unique CDR3 sequences at late time points also confirmed that our repertoire analysis was highly constricted to the antigen-specific pool as, e.g., sort impurities would have led to sequencing of random clonotypes and thereby continuously increased the number of overall unique CDR3 sequences (Figure 1c).

### 2.2. Public Clonotypes Lead to Repertoire Saturation across Individual Mice

**Figure 2:**
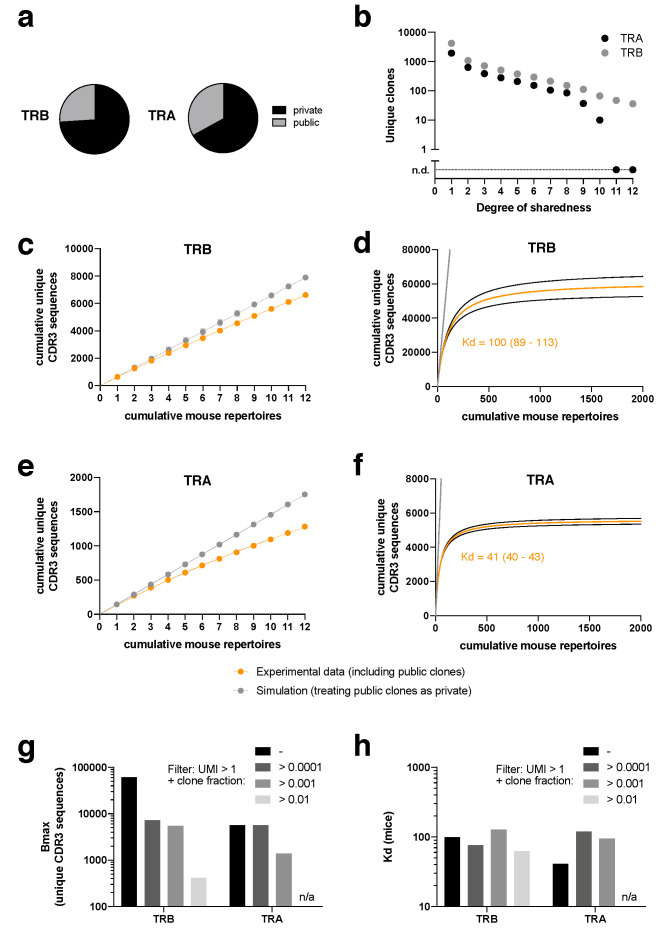
Cumulative absolute size of H2k^b^/SIINFEKL-specific TCR repertoires (**a**) Share of public clonotypes; (**b**) unique CDR3 sequences for different degrees of sharedness (maximum degree of sharedness 12/12); (**c**) cumulative unique CDR3β sequences vs. cumulative mouse repertoires; orange denotes experimental data including public clones; gray denotes simulated outcomes if all public clones are treated as private clones; (**d**) function of fitted data from (**c**); black lines surrounding orange line indicates 95% confidence interval; Kd denotes number of mice at which half of the maximum cumulative CDR3 sequences would be observed; (**e**) as in (**c**), but for CDR3α; (**f)** as in (**d**), but for CDR3α. (**g**) Bmax (number of unique CDR3 sequences when X equals an infinite number of cumulative mouse repertoires) for CDR3β and CDR3α in dependence of clone fraction filters. All clones had a filter of UMI > 1. (**h**) as in (**g**), but for Kd (number of mice to reach 50% of Bmax). All data come from non-thymectomized animals.

Of all unique clonotypes, about a third of CDR3α and a quarter of CDR3β sequences were “public”, which means that they occurred in more than one mouse at any time point (Figure 2a). In addition to the mere information whether a clonotype was public or not, our standardized repertoire analysis in at least 12 mice per treatment allowed us to investigate the degree of sharedness. While 1939 unique CDR3α and 4225 unique CDR3β sequences were private, we also found CDR3α and CDR3β sequences across the entire spectrum of sharedness, including 10 CDR3α sequences found in 10/12 non-thymectomized mice and 36 unique CDR3β sequences found in 12/12 non-thymectomized mice (Figure 2b).

These data indicate that the overall TCR repertoire against SIINFEKL is diverse but limited. The presence of extreme “supra-public” clonotypes prompted us to investigate whether we could observe saturation effects when adding individual mouse repertoires cumulatively (Figure 2c–f). If there were no public clones, with each added mouse repertoire the number of cumulative unique CDR3β (Figure 2c,d) or CDR3α (Figure 2e,f) sequences would rise infinitely without saturation (gray data points and lines indicate an according simulation, treating public clones from the experimental data as private). Since in the experimental data we do see a substantial degree of sharedness, the cumulative numbers of unique CDR3 sequences start to fall below the “private only simulation diagonal” already within the 12 investigated TCR repertoires (Figure 2c,e). We wondered whether we could make an extrapolation from these data to obtain a ballpark figure at which cumulative number of unique CDR3 sequences there would be a plateau, provided an infinite number of mice were investigated (Figure 2d,f). To this end, we fitted the data to a simple saturation equation, Y = Bmax*X/(Kd + X), in which Y is the cumulative number of unique CDR3 sequences and X is the number of cumulative mouse repertoires. Bmax then indicates the saturated number of unique CDR3 sequences if an infinite number of mice was studied and Kd indicates the number of mice that is needed to observe 50% of Bmax. With this approach, we would observe saturation at a little less than 60,000 CDR3β sequences and a little less than 6000 CDR3α sequences. While 100 mice (=Kd) would need to be studied to see half of the overall maximum of CDR3β sequences, 41 mice would need to be studied in case of CDR3α. It is important to stress that the exact numbers should not be overinterpreted as our approach is prone to several confounding factors. While we did use UMI in our sequencing approach (reducing the likelihood for sequencing error bias), sequencing from peripheral blood is inherently subject to sampling bias. Furthermore, we did not perform αβ pairing analysis and our PCR was based on a RACE PCR protocol, making abundance estimates from bulk sequencing data unprecise, which would however be important for precise extrapolation of “unseen species”. These considerations are well-described challenges [19,21,32,33]. Throughout our analyses, we only analyzed clonotypes with a UMI > 1. When we set additional filters with threshold clone fractions (i.e., when we only analyzed clones that showed a minimum clone fraction), Bmax decreased with increasingly strict filters (Figure 2g). Remarkably, Kd remained rather stable (Figure 2h).

Overall, the data do allow us to draw two general conclusions: first, the number of unique clonotypes for H2k^b^/SIINFEKL is highly diverse, but limited to an extent that makes reliable estimation of absolute numbers feasible for future investigation. Second, the number of unique SIINFEKL-specific CDR3α sequences is lower than the number of unique CDR3β sequences.

### 2.3. (Supra-) Public Clonotypes Are Preferentially Expanded during the Immune Response

**Figure 3:**
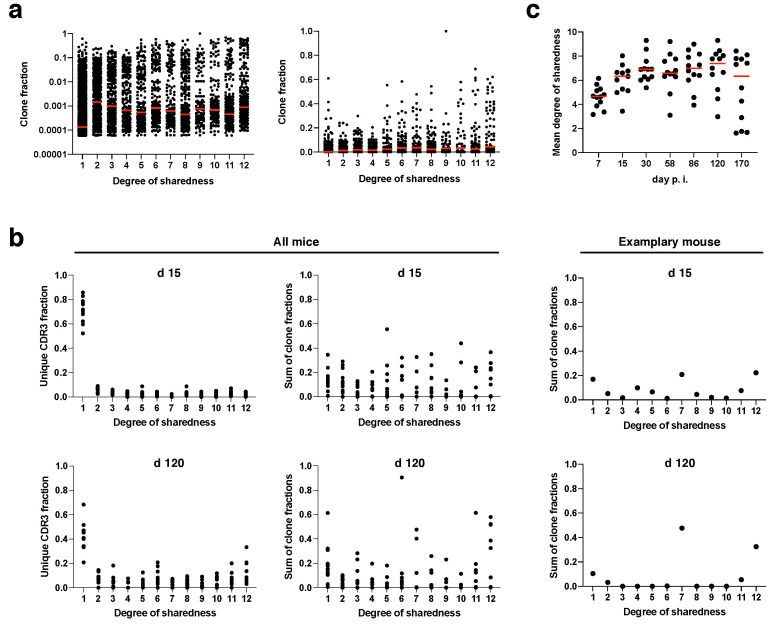
Clone fractions in dependence of degree of sharedness. (**a**) Clone fraction of CDR3β sequences for different degrees of sharedness (maximum degree of sharedness 12/12; data from all mice and time points pooled); left: log scale; right: linear scale. (**b**) fraction of unique CDR3β sequences (left column) or sum of clone fractions (middle and right columns) for all mice (left and middle column) or one representative mouse (right column) vs. the degree of sharedness for day 15 (top) or day 120 (bottom) after infection; sum of clone fraction means all clone fractions with a specific degree of sharedness were added up; each dot indicates data for one degree of sharedness in one mouse; (**c**) mean degree of sharedness over time post infection; each dot represents one mouse repertoire for a given time point; all data come from non-thymectomized animals.

Next, we investigated whether public clones are also preferentially recruited into the immune response. While evidence for this has been collected before, previous analyses were mostly confined to individual time points and investigated only private vs. public clones in general [15,16,25], thereby not incorporating the resolution of differential degrees of sharedness over the entire course of an immune response. Since the CDR3β repertoire is more diverse than the CDR3α repertoire and at the same time less prone to bias through second chains from non-excluded alleles, we focused our analyses from hereon on CDR3β sequences. We first looked at all clonotypes from all time points and mice pooled to obtain a broad overview (Figure 3a). The median clone fraction (i.e., the fraction to which a clone contributes to the antigen-specific T cell response in a given mouse and at a given time point) of public clonotypes (degree of sharedness > 1) was generally about one order of magnitude higher than the median clone fraction of private clonotypes (degree of sharedness = 1). While the median clone fraction did not differ further among different degrees of sharedness, individual clones with a clone fraction > 0.2 mostly had a degree of sharedness of at least 5/12. Particularly supra-public clones with a degree of sharedness of 12/12 massively contributed to the overall immune response. Next, we concatenated the clonotypes with a given degree of sharedness for each mouse, and investigated the fraction of all unique CDR3β sequences and the sum of all clone fractions that would be represented by clones with a given degree of sharedness at day 15 and at day 120 post infection (Figure 3b). While private clones constituted about 70% of all unique CDR3β sequences (Figure 3b, top left panel), they only contributed about 15% to the overall clone fraction at day 15 post infection (Figure 3b, top right panel). Supra-public clonotypes with a degree of sharedness of 12/12 in turn contributed about 15% to the overall clone fraction, but only constituted 2% of unique CDR3β sequences to begin with. These data indicate preferential expansion of public clonotypes (in particular supra-public clonotypes with extreme degrees of sharedness).

Compared to day 15 post infection, at day 120 private clonotypes constituted less unique CDR3β sequences (Figure 3b, bottom panels). This indicates that during memory inflation, public clones are not only more likely to expand, but also more likely to be detectable at all. Next, we plotted the mean degree of sharedness of individual mouse repertoires over time (Figure 3c). Due to preferential expansion of (supra-)public clonotypes, the mean degree of sharedness was at around 5/12 already at day 7 post infection. The mean degree of sharedness rose to a little more than 6/12 from day 15 post infection onwards. In individual mice, the mean degree of sharedness fell again at late time points of infection due to the fact that, by chance, stable dominators were selected during memory inflation which showed partly low degrees of sharedness (see Figure 1d and [29] for illustration of stable dominators after day 58 post infection). Across all mice the median of the mean degree of sharedness was rather stable at a remarkably high value (Figure 3c).

### 2.4. TCR Generation Probability Is Linked with Degree of Sharedness and Thereby Clonal Expansion

**Figure 4:**
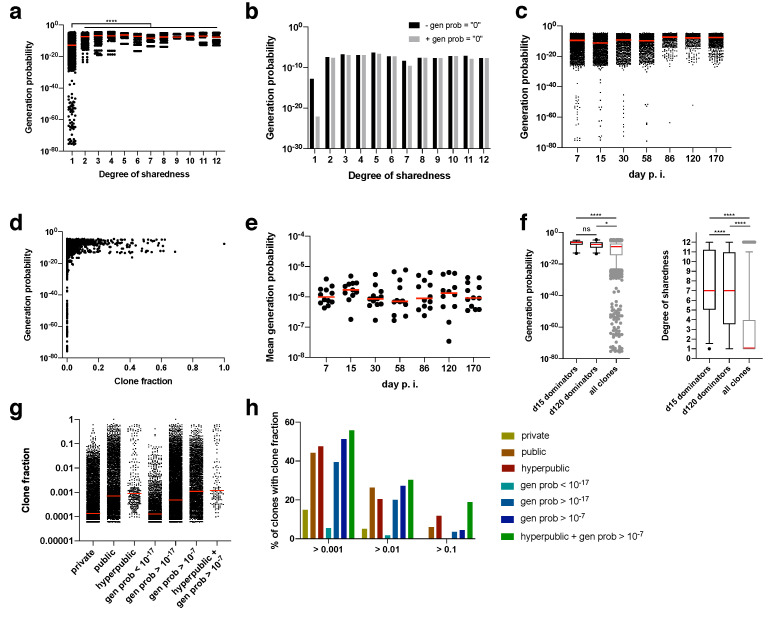
Association of generation probabilities with degree of sharedness and clone fraction. (**a**) Generation probabilities of CDR3β sequences for different degrees of sharedness (maximum degree of sharedness 12/12; data from all mice and time points pooled); clonotypes for which no generation probability could be calculated were not included in this depiction; medians are shown in red. (**b**) medians from (**a**) without (black) or with (gray) clonotypes for which no generation probability could be calculated (gen prob = “0”); (**c**) as in (**a**), but over time post infection, statistical testing by Kruskal-Wallis test (****) followed by Dunn’s multiple comparisons test (shown are results for comparisons of each day from day 86 onwards tested against each day until day 58); **** *p* value < 0.0001; (**d**) as in (**a**), but against clone fraction at a given time point in a given mouse; (**e**) mean generation probability over time post infection; each dot represents one mouse repertoire for a given time point; (**f**) generation probabilities for dominators (i.e., clones with a clone fraction >0.1) at day 15 and day 120 post infection, in comparison to all clones; medians in red, boxes indicate 95% confidence interval; statistical testing by Kruskal-Wallis test (****) followed by Dunn’s multiple comparisons test; ns non-significant, * *p* value < 0.05, **** *p* value < 0.0001; (**g**) Clone fraction of clones with specified filters from all mice and time points pooled; (**h**) data from (**g**), but percentage of clones with a clone fraction above the indicated values (below the bars) depending on the filters (shown on the right); all data come from non-thymectomized animals.

Differential generation probabilities of TCR sequences could be an explanation for differential degrees of sharedness. We therefore calculated generation probabilities for each amino acid clonotype using the OLGA algorithm (taking into account, among others, preferential VDJ pairing, the number of required insertions and deletions and motifs) [26]. Clonotypes with extremely low generation probabilities (<10^−25^) were found exclusively for private clonotypes (Figure 4a). Since there are also more private than public clonotypes, we calculated the median generation probability. Irrespective of whether clonotypes were included for which no generation probability could be calculated (presumably, but not necessarily, because it was too low; gen prob = “0”), the median generation probability rose from private to public clonotypes by several orders of magnitude (Figure 4a,b). Nearly all of the clonotypes for which no generation probability could be calculated were private, which explains why the median shifted the most for private clonotypes (Figure 4b).

During the course of the immune response, clones with extremely low generation probabilities were lost (Figure 4c). Also, clones with a minimum degree of clone fraction showed a threshold generation probability. Clones with a clone fraction of > 0.1 at any time point had a minimum generation probability of 10^−17^ (Figure 4d). Since mostly clones with a high generation probability expanded, the mean generation probability was as high as 10^−6^, which did not change over time (Figure 4e). This was further confirmed by focusing on the dominating clones at the peak of the acute immune response (day 15 post infection) and during memory inflation (day 120 post infection). The dominating clones (i.e., clones with a clone fraction > 0.1) had exclusively very high generation probabilities (Figure 4f). These high generation probabilities led in most cases to very high degrees of sharedness (median of 7 for day 15 and day 120 post infection). In turn, clones with a high degree of sharedness and/or generation probability represented higher median clone fractions (Figure 4g). We therefore finally explored whether generation probability and degree of sharedness could serve as predictors of TCRs that clonally expand within an antigen-specific immune response. In fact, both generation probability and degree of sharedness independently selected for clones with a higher clone fraction, with the combination of both parameters being the filter with the highest enrichment of expanding clones (Figure 4h). Among supra-public clonotypes with a generation probability of 10^−7^ there was a 18.8% hit rate of clones with a clone fraction > 0.1. This hit rate is 0.2% without any filter, indicating a 94-fold enrichment of clones that will at some point contribute more than 10% (clone fraction of > 0.1) to an overall antigen-specific T cell response.

### 2.5. The Nucleotide-to-Amino Acid Ratio of Intraindividual TCR Sequences Is Associated with Interindividual Degrees of Sharedness via Generation Probability

It was previously speculated that the TCR generation machinery could have evolved in a manner that TCRs with high functionality against foreign epitopes are preferentially generated [27]. This hypothesis is intriguing, but hard to confirm experimentally. An alternative explanation why TCRs with high generation probability are not only shared among many different individuals, but also robustly expand, is that high generation probabilities lead to repeated generation of the same clonotype within a given individual, thereby increasing the precursor frequencies of antigen-specific T cells in the naïve repertoire. We have previously shown how tightly increased precursor frequencies are linked to the robustness of antigen-specific T cell expansion [34]. For CDR3 nucleotide sequence copies it is not possible to deduct whether these sequences are derived from true clones (progeny of the same single cell) or if, by chance, the same nucleotide sequence has been generated twice. Functionally, however, TCRs are only determined through their amino acid sequence and due to the degeneracy of the base triplet code, the same CDR3 amino acid sequence can be generated through different nucleotide sequence variants (in fact, such convergence of different nucleotide sequences into one CDR3 amino acid sequence is an important parameter of the computation of CDR3 sequence generation probabilities [26]).

**Figure 5:**
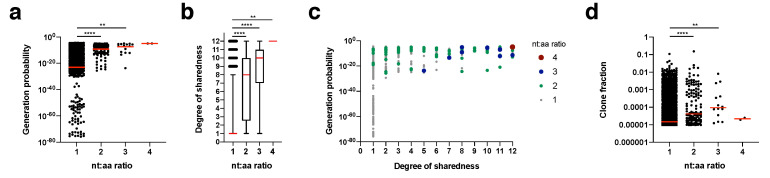
Association of nucleotide-to-amino acid ratio of TCR sequences to generation probability and degree of sharedness. (**a**) Generation probability of CDR3β sequences (all time points and mice pooled) vs. the nucleotide-to-amino acid (nt:aa) ratio found in individual mice; (**b**) as in (**a**), but for degree of sharedness and with boxes indicating 95% confidence interval and individual data points displayed outside this interval; (**c**) as in (**a**,**b**), but with all data in one graph; (**d**) as in (**a**), but against clone fraction; all data come from non-thymectomized animals; red line indicates median; statistical testing for all data by Kruskal-Wallis test (****) followed by Dunn’s multiple comparisons test; ** *p* value < 0.01, **** *p* value < 0.0001.

We calculated for each clonotype how many nucleotide sequence variants lead to the same amino acid sequence (nt:aa ratio) within an individual mouse (Figure 5). This nt:aa ratio could be indicative of the precursor frequency for an amino acid clonotype within a given mouse. Generation probability (Figure 5a) and degree of sharedness (Figure 5b) were both strongly associated with the nt:aa ratio (see Figure 5c for integrated data analysis). A single clonotype (CASSRTGEQYF) appeared in the form of four different nucleotide sequence variants (nt:aa ratio = 4) in two mice. This clone had one of the highest generation probabilities (1.04^−5^) and a maximum degree of sharedness (12/12). Clonotypes with a nt:aa ratio of 3, 2 or 1 had median generation probabilities and degrees of sharedness in descending respective orders. We finally investigated whether we could also directly observe that heightened nt:aa ratios would result in higher clone fractions. Indeed, median clone fractions rose with increasing nt:aa ratios from 1 to 3. For the two instances in which the clonotype CASSRTGEQYF was observed in the form of four different nucleotide sequence variants, it did not reach a high clone fraction. Overall, these data suggest that increased precursor frequencies, as potentially indicated by heightened nt:aa ratios, are not only a function of increased generation probabilities, but also result in higher degrees of sharedness. On average (as expressed by the median), higher nt:aa ratios result in higher clone fractions, but more and more precise data are needed to conclusively address this.

## 3. Discussion

We sequenced the TCR repertoire of IE2-OVA-mCMV-specific T cell responses in-depth over time. While we have previously focused our analysis on the successive outgrowth of dominating clones [29], in this work we first investigated how many new unique clones are recruited during the course of the immune response. This revealed that the TCR repertoire stabilizes during memory inflation not only in terms of its evenness, but also in terms of its composition of unique clonotypes. In non-thymectomized animals, stabilization occurred at a little less than 400 unique CDR3β sequences per mouse. This number is likely an underestimate of the absolute number of different clonotypes, since we cannot deduct with how many different CDR3α chains these CDR3β chains are pairing and the analysis from peripheral blood will be subject to sampling bias. These challenges to estimate the absolute number of unique TCRs, many of which are analogous to the “unseen species problem” in ecology, are extensively discussed elsewhere [21,32,35,36]. Of note, however, the CD8+ T cell precursor frequency for SIINFEKL has previously been reported to be 50–200 cells [37] and our own observations suggest this number to be even slightly higher (unpublished data). These numbers should approximate the number of overall observed sequences in the memory repertoire—if recruitment into the immune response is as complete as it has been reported for OT-I cells [38]—and therefore render some credence to our obtained numbers of unique TCRs per mouse.

Independent from these considerations, the observed repertoire stabilization during memory inflation is striking. First, this confirmed that our sequencing methodology was technically clean, as e.g., sorting impurities would have led to capturing random sequences and therefore to a continuous increase in cumulative unique CDR3 sequences. Second, while we most probably missed TCR sequences due to sampling bias at early time points, at least at late time points the sampling bias did not seem to be a confounding factor. Third, from a biological perspective nourishment of the antigen-specific pool during memory inflation did not seem to come from novel thymic emigrants. This is in line with previous reports that inflationary T cells are mainly fed by early-primed memory T cells [31]. In thymectomized animals the cumulative number of unique CDR3 sequences was reduced. The fact that this reduction became apparent only at later time points could be explained by a more dominant sampling bias at early time points, although a role of thymic emigrants contributing to the late immune response cannot be excluded [31]. Fourth, the saturation of repertoires within individual mice gave us a leverage point to look at saturation across individual mice.

Such a saturation across individual mice is only possible when clonotypes are public. We confirmed previous reports that many CMV-specific clonotypes occur in more than one individual organism [15,16,17]. In fact, we observed that the number of cumulative unique CDR3 sequences did not rise in a linear manner with increasing cumulative mouse repertoires analyzed, suggesting saturation to occur at some point. We applied a saturation model to our data to more closely investigate this. We observed that the maximum antigen-specific repertoire should be highly diverse, but limited, and predict that half of the maximum of overall TCR sequences should be visible at around 100 cumulative mouse repertoires, with the exact numbers being confounded by the same biases as discussed above. Future studies may therefore be able to more accurately predict the absolute number of unique clonotypes in a mouse by investigating around 100 mice (due to the present biases, this number may vary, but it is unlikely that it will be an underestimate by several orders of magnitude) while overcoming confounding factors by single-cell sequencing and harvesting material from blood and all lymphoid organs.

We observed that public clonotypes constituted the majority of expanded clones despite accounting only for a minority of unique CDR3 sequences, which is in line with previous reports investigating this aspect for IE3- and M38-specific T cell responses at late time points of infection [25]. Beyond mere classification into private vs. public, the degree of sharedness can be highly heterogenous. Few studies have so far investigated the degree of sharedness in a systematic manner [23] despite its importance for contextualization of public sequences in respect to their generation probabilities [20]. We observed a considerable number of clonotypes in up to 10/12 mice for CDR3α and 12/12 mice for CDR3β. These “supra-public” clonotypes often dominated the antigen-specific response, which is completely disproportionate given their rarity (meaning how few of all unique CDR3 sequences they represent). Overall, the mean degree of sharedness of the antigen-specific repertoire was in-between 5–7 out of 12 mice, with no major shifts over time. To the best of our knowledge, this study is the first to perform a systematic and in-depth investigation these aspects for CMV, but also any kind of antigen-specific immune response.

The occurrence of public TCR sequences is tightly associated with TCR generation probability [20,22]. We applied OLGA [26] for accurate estimation of generation probability to our unique data set of TCRs covering a wide range of degrees of sharedness and defined time points after infection. Generation probability markedly influenced the likelihood for a TCR to be public (with a threshold of at least 10^−17^), but did not further predict the degree of sharedness. The latter stands in contrast to previous reported work, which however only investigated TCR sequences in bulk (and did not focus on certain antigen-specificities) and thereby also analyzed a much larger number of sequences [20]. We hypothesize that, apart from sampling bias, TCR affinity could be a relevant confounding factor in this regard [18]. Overall, dominating clones at the acute phase of the immune response and during memory inflation showed high degrees of sharedness as well as high generation probabilities and we could show that the combination of both parameters can serve as an enrichment filter for identifying clones that will contribute significantly to the antigen-specific T cell response (arguably representing highly functional TCRs). This may prove useful for in silico prediction of TCRs with clinical potential in the context of adoptive cell therapy.

It has been argued that the TCR recombination machinery may have evolved in a manner that highly functional TCRs are inherently more probable to be generated [27]. As intriguing as this thought is, such evolutionary aspects are hard to investigate experimentally. Instead, we hypothesize that heightened intraindividual precursor frequencies may explain why TCRs with high generation probability expand more robustly. To this end, we used the intraindividual number of nucleotide sequence variants that give rise to one common TCR amino acid sequence (nt:aa ratio) as a proxy indicator of precursor frequencies within a given mouse. Both generation probability and degree of sharedness were tightly associated with the nt:aa ratio. Since theoretical nt:aa ratio possibilities for a given sequences are an inherent part of generation probability calculation, this is not surprising, but validates the calculation and confirms the tight association with the degree of sharedness. On average (as expressed by the median), an elevated nt:aa ratio also indicated a higher clone fraction, although more data are needed to corroborate this point. A high clone fraction in clones with a nt:aa ratio of 1 does not exclude the possibility of an increased precursor frequency since, naturally, high generation probabilities can also lead to the exact same nucleotide sequence being generated within one organism repetitively. We have previously shown how much the robustness of a clonal response depends on the number of precursors [34]. The exact copy number abundance of individual clonotypes that is present in a naïve antigen-specific repertoire therefore represents a highly relevant point for future investigation.

Overall, here we show that generation probability is a major determinant of the T cell response to SIINFEKL-expressing CMV. The results are consistent with so far described T cell responses against other natural CMV epitopes [25], arguing against a special intrinsic feature of SIINFEKL to induce (supra-)public T cell responses. Instead, the fact that similarly public responses are observed towards natural CMV epitopes points into the direction that the public nature of the T cell response could be due to the mode of antigen presentation rather than the epitope itself. This is in line with reports describing that it is rather the context of gene expression that defines the immunodominance hierarchy of CMV antigens [30]. In future studies, it will be important to investigate both T cell responses against the same epitope under different expression modes as well as against different epitopes (ideally expressed in the same mode) to shed further light on these issues. Since many different model systems exist for SIINFEKL, our work provides a resource to compare the T cell responses to the same epitope under different expression systems (e.g., by using *Listeria monocytogenes* OVA or OVA-expressing tumor model systems). Such comparisons will be particularly informative since CMV vectors are being investigated for vaccine delivery, and CMV epitope presentation seems to induce unconventional immunogenicity patterns [39]. Our choice to study the T cell response against a model antigen in a highly controlled experimental system was therefore deliberate. It should be noted, however, that translation of our findings to the human setting will also be complicated by the fact that human beings are highly diverse in their HLA status (which has a major effect on the immunodominance of individual epitopes) and can be re-infected by different CMV strains.

While we have previously reported TCR affinity to be a significant modulator of T cell fate during CMV infection [29], we did not observe such dramatic changes with regards to the mean generation probability over time (during the course of the immune response, e.g., when comparing the peak of the acute response to time points during memory inflation). Rather, it appears that generation probability affects the likelihood to significantly expand within the T cell response at any time point during infection. We therefore hypothesize that most of the presented results are transferrable to other infection and perhaps even non-infectious disease settings.

## 4. Materials and Methods

### 4.1. Experimental Data

All experimental data have been previously published [29]. In brief, mice were thymectomized or non-thymectomized at the age of four weeks. Completeness of thymectomy was controlled by macroscopic inspection, and an effect on thymic output was controlled for by qPCR for TREC. The operating procedure did not result in any confounding effects as controlled for through mock operated cohorts in pilot experiments (unpublished data). C57BL/6 mice were infected with an mCMV strain expressing OVA under the IE2 promotor [24] at the age of seven weeks. All mice were distributed across two independent experimental cohorts (two non-thymectomized cohorts, two thymectomized cohorts). We sorted the H2k^b^/SIINFEKL-specific CD8+ T cell population by flow cytometry at day 7, 15, 30, 58, 86, 120 and 170 post infection with sort purities most often reaching 100% [23]. For CDR3 sequencing, RNA of sorted antigen-specific T cells was reverse-transcribed into cDNA using a biotinylated oligo dT primer. An adaptor sequence was added to the 3’ end of all cDNA, which contains the Illumina P7 universal priming site and a 17-nucleotide UMI. Products were purified using streptavidin-coated magnetic beads followed by a primary PCR reaction using a pool of primers targeting the TCRα and TCRβ regions, as well as a sample-indexed Illumina P7C7 primer. The TCR-specific primers contained tails corresponding to the Illumina P5 sequence. PCR products were then purified using AMPure XP beads. A secondary PCR was performed to add the Illumina C5 clustering sequence to the end of the molecule containing the constant region. The number of secondary PCR cycles was tailored to each sample to avoid entering plateau phase, as judged by a prior quantitative PCR analysis. Final products were purified, quantified with Agilent Tapestation and pooled in equimolar proportions, followed by high-throughput paired-end sequencing on the Illumina MiSeq platform. For sequencing, the Illumina 600 cycle kit was used with the modifications that 325 cycles was used for read 1, 6 cycles for the index reads, 300 cycles for read 2 and a 20% PhiX spike-in to increase sequence diversity.

### 4.2. Clone Identification

Extraction and quantification of T-cell receptor sequences was performed using IGX-Profile software (ENPICOM B.V., ‘s-Hertogenbosch, The Netherlands). Sequences were considered only if (1) an intact UMI barcode was present and (2) the UMI was found more than once in a given sample. Clonotypes were defined as chains with identical CDR3 amino acid sequences. Receptor counts were summed for sequences with differing CDR3 nucleotide sequences, provided they shared CDR3 amino acid identity. Samples with exceptionally low read counts (<45,000 reads) were excluded from analysis. Receptors were further filtered if one of the following criteria was met: (1) Average PHRED quality score was below 20; (2) CDR3 sequence contained a stop codon; (3) CDR3 was shorter than 6 amino acids long.

All data grouping, merging and processing was performed with Python 3, using the Pandas library.

### 4.3. Generation Probability

Calculation of the generation probability was based on the CDR3 amino acid sequence. For this, OLGA [26] was used. The generation probability was calculated for TRB (CDR3β) sequences. The generation probability was not calculated for TRA (CDR3α) due to the lack of availability of model parameters. Sequences with a generation probability of 0 were excluded from analysis unless otherwise indicated.

For the saturation analysis, the clones belonging to the same mouse were pooled. Next, a random ordering of mice (e.g., [mouse_{1}, mouse_{5}, … mouse_{2}]), was iterated. At each step of the iteration, the set of clones found in a mouse was merged with the set of all previously observed clones. The result was a set of data points mapping the number of mice (=iteration number) to the number of unique clones observed. 100 such random orderings were made, and the resulting data points were then fitted using a saturation model. For this, the following equation was used:

Y = Bmax × X/(Kd + X)

in which Y is the cumulative number of unique CDR3 sequences and X is the number of cumulative mouse repertoires. Bmax then indicates the saturated number of unique CDR3 sequences if an infinite number of mice was studied, and Kd indicates the number of mice that is needed to observe 50% of Bmax. Fitting was performed with Prism GraphPad (Version 8.1.2).

### 4.4. Statistical Analyses

Statistical analyses were performed using Prism GraphPad (Version 8.1.2) software. Statistical tests were used as indicated.
